# Handheld laser-fiber vibrometry probe for assessing auditory ossicles displacement

**DOI:** 10.1117/1.JBO.26.7.077001

**Published:** 2021-07-21

**Authors:** Marcin Masalski, Adam Wąż, Przemysław Błauciak, Tomasz Zatoński, Krzysztof Morawski

**Affiliations:** a Wroclaw Medical University, Department of Otolaryngology Head and Neck Surgery, Wroclaw, Poland; b Wroclaw University of Science and Technology, Department of Biomedical Engineering, Wroclaw, Poland; c Wroclaw University of Science and Technology, Department of Field Theory, Electronic Circuits, and Optoelectronics, Wroclaw, Poland; d Wroclaw Medical University, Department of Neurosurgery, Wroclaw, Poland; e University of Opole, Institute of Medical Sciences, Opole, Poland

**Keywords:** laser-fiber vibrometer, handheld probe, middle ear mechanics, middle ear surgery, laser-Doppler vibrometer

## Abstract

**Significance:** Measurements of auditory ossicles displacement are commonly carried out by means of laser-Doppler vibrometry (LDV), which is considered to be a gold standard. The limitation of the LDV method, especially for *in vivo* measurements, is the necessity to expose an object in a straight line to a laser beam operating from a distance. An alternative to this approach is the use of a handheld laser-fiber vibrometry probe (HLFVP) with a curved tip.

**Aim:** We evaluate the feasibility of an HLFVP with a curved tip for measuring sound-induced displacement of the auditory ossicles.

**Approach:** A handheld vibrometer probe guiding the laser beam with a fiber-optic cable was used for displacement measurements of the incus body and the posterior crus of the stapes. Tonal stimuli at frequencies of 0.5, 1, 2, and 4 kHz were presented by means of an insert earphone positioned in the outer ear canal. The probe was fixed at the measurement site using a tripod or hand-held by one of the two surgeons.

**Results:** The measurements were carried out on six fresh temporal bones. Multivariate analysis of variance showed statistically significant differences for stimulus frequency ($F_{3,143}=29.37$, p<0.001, and $\eta^{2}=0.35$), bone ($F_{5,143}=4.61$, p=0.001, and $\eta^{2}=0.01$), and measurement site ($F_{1,143}=4.74$, p=0.03, and $\eta^{2}=0.02$) in the absence of statistically significant differences for the probe fixation method ($F_{2,143}=0.15$, p=0.862, and $\eta^{2}=0.001$). Standard deviations of the means were 6.9, 2.6, 1.9, and 0.6  nm/Pa for frequency, bone, site, and fixation, respectively. Ear transfer functions were found to be consistent with literature data.

**Conclusions:** The feasibility of applying HLFVP to measure the displacement of auditory ossicles has been confirmed. HLFVP offers the possibility of carrying out measurements at various angles; however, this needs to be standardized taking into account anatomical limitations and surgical convenience.

## 1 Introduction

Laser-Doppler vibrometry (LDV) is widely used in the assessment of auditory ossicles displacement. The method has been used in fundamental research on middle ear mechanics,^1^^–^^6^ optimization of surgical procedures^2^^,^^7^^–^^15^ such as intraoperative qualification for implantable middle ear hearing devices,^2^^,^^16^^,^^17^ and intraoperative evaluation of hearing improvement during ossiculoplasty.^18^^–^^26^ Vibrometric studies have also been conducted for potential applications in the clinical diagnosis of hearing loss.^27^^–^^30^ The LDV method enables non-contact measurement in a wide frequency band at a precisely determined point and is a gold standard in cadaver tests. However, despite numerous *in vivo* studies, it is not routinely used in clinical settings.

One of the LDV’s limitations is the requirement to access the measurement site in a straight line. This often prevents reaching middle ear elements without damaging functionally relevant structures, especially since the laser beam must also bypass the eardrum, which collects the sound stimulus. In particular, these limitations apply to the measurement of the stapedial footplate, which is the last element of the middle ear’s conductive chain. With a standard surgical approach to the middle ear cavity via a posterior tympanotomy, the access to the footplate is often limited by the facial nerve canal. In cadaver studies, the approach is extended by removing the canal and opening the facial recess,^7^^,^^8^^,^^11^^,^^31^^,^^32^ which cannot be done *in vivo* due to facial nerve palsy. *In vivo* measurement must be carried out on the posterior crus of the stapes^1^^,^^3^^,^^15^^,^^17^^,^^24^^,^^31^ or prosthesis in the case of crus damage.^19^^–^^22^ The transcanal approach consisting in elevation of the tympanic membrane is possible for vibrometry measurement using an alternative electromagnetic stimulation by means of a coil placed on the manubrium of the malleus.^10^^,^^23^ Nonetheless, the measuring site must still be within a straight line to the LDV device.

The development of endoscopic middle ear surgery techniques offering wider views and access to the areas of middle ear unreachable by microscopes^33^^–^^36^ entails the need to develop a vibrometric probe that can overcome the limitations of the straight-line access to the measurement site. Additionally, due to the high costs of LDV, alternative solutions in the form of handheld probes have been investigated.^37^^–^^42^ Handheld probes measure the displacement of movable tips that are excited by touching the measurement site. Displacement can be registered by piezoelectric sensors,^37^^,^^40^ strain gauge,^39^ electromagnetic,^38^ electromechanical,^42^ or fiber-optic Fabry–Perot strain sensors.^41^ The general problem of touch probes is tremor leading to high pretension and stiffening of the measuring site. This phenomenon requires the use of additional control systems, e.g., hydraulic^39^ or magnetic ones.^42^

This paper presents a handheld laser-fiber vibrometry probe (HLFVP) that guides the laser beam to the measurement site with a fiber-optic cable, thus allowing the access to spots unreachable by the laser beam of standard LDV device, while maintaining the advantages of non-contact measurement.^43^ The aim of this study is to evaluate the feasibility of using the HLFVP in the measurement of the auditory ossicles displacement and to compare the results with data reported in the literature.

The concept of the HLFVP is to illuminate a vibrating object with a laser beam coming out of an optical fiber and to collect diffused laser beam shifted in the frequency domain by the Doppler effect to a second optical fiber. The displacement is calculated based on the interference of the diffused laser beam with the reference beam by means of phase detection and demodulation. Standard LDV uses He–Ne gas lasers with a wavelength of 632.8 nm, which is subject to significant limitations when guided by an optical fiber. The use of the semiconductor laser diode with a wavelength of 1550 nm allowed for applying fiber-optic technology to developing the laser-fiber Doppler vibrometer. Since the light at a wavelength of 1550 nm is invisible to the human eye, a navigation spot of red light is introduced to mark the site.^43^

Displacement measurements depend on the site and approach to the site, which is directly related to the measurement angle between the laser beam and the direction of movement.^3^^,^^31^ Relatively small differences are related to the type of material: *in vivo*, fresh, or frozen temporal bones.^44^^,^^45^ To compare the study results with the data available in the literature,^1^^–^^3^^,^^12^^,^^16^^,^^17^^,^^31^ measurements were performed on the posterior crus of the stapes and the incus body using the surgical approach via posterior tympanotomy with the facial nerve preservation. In this study, fresh cadaver specimens were used.

## 2 Materials and Methods

The study was carried out in the Dissection Room of the University Clinical Hospital during the anatomopathological examination. The consent to conduct the trial was granted by the Bioethics Committee of Wroclaw Medical University in accordance with the World Medical Association Declaration of Helsinki. Each time, the consent to the anatomopathological examination was issued according to the applicable legal basis.

### 2.1 Material

Tests were performed on cadaveric donors within 48 h of death. The cadaver was kept in a cold storage at 4.5°C and placed on a sectional table about 3 h prior to the measurements. The external auditory canal was examined otoscopically for the presence of a normal eardrum. Cerumen was removed if present. The ER-3A insert earphone (Etymotic Research, IL, USA) with foam ear-tip was placed in the external acoustic canal. After the behind-the-ear incision, a self-retaining Weitlaner retractor was positioned to hold the auricle forward, and the mastoid plane was exposed. A standard antromastoidectomy followed by posterior tympanotomy with preservation of the facial nerve canal and the chorda tympani was performed. The stapedius tendon was severed to improve access to the posterior crus of the stapes. Measurements were conducted directly after preparation.

### 2.2 Sound Stimulus

Tonal stimuli at frequencies of 500 Hz, 1, 2, and 4 kHz were presented simultaneously using earphones driven by a programmable generator RIGOL DG1062Z (Rigol, OR, USA). The voltage on the generator was adjusted to match the voltage of the Interacoustic AD629 (Interacoustic, Denmark) audiometer calibrated in accordance with ISO 389-1:1998 for the same headphones as used in the measurements. The sound pressure level at the eardrum was determined based on the real-ear to dial difference (REDD) values^46^ (Table 1).

**Table 1:** REDD values for ER-3A earphones with foam ear-tip by Munro and Lazenby.^46^

Frequency (Hz)	500	1000	2000	4000
REDD (dB) (SD)	9.6 (1.6)	5.7 (1.8)	11.9 (1.7)	8.8 (1.9)

### 2.3 Measurements

**Fig. 1:**
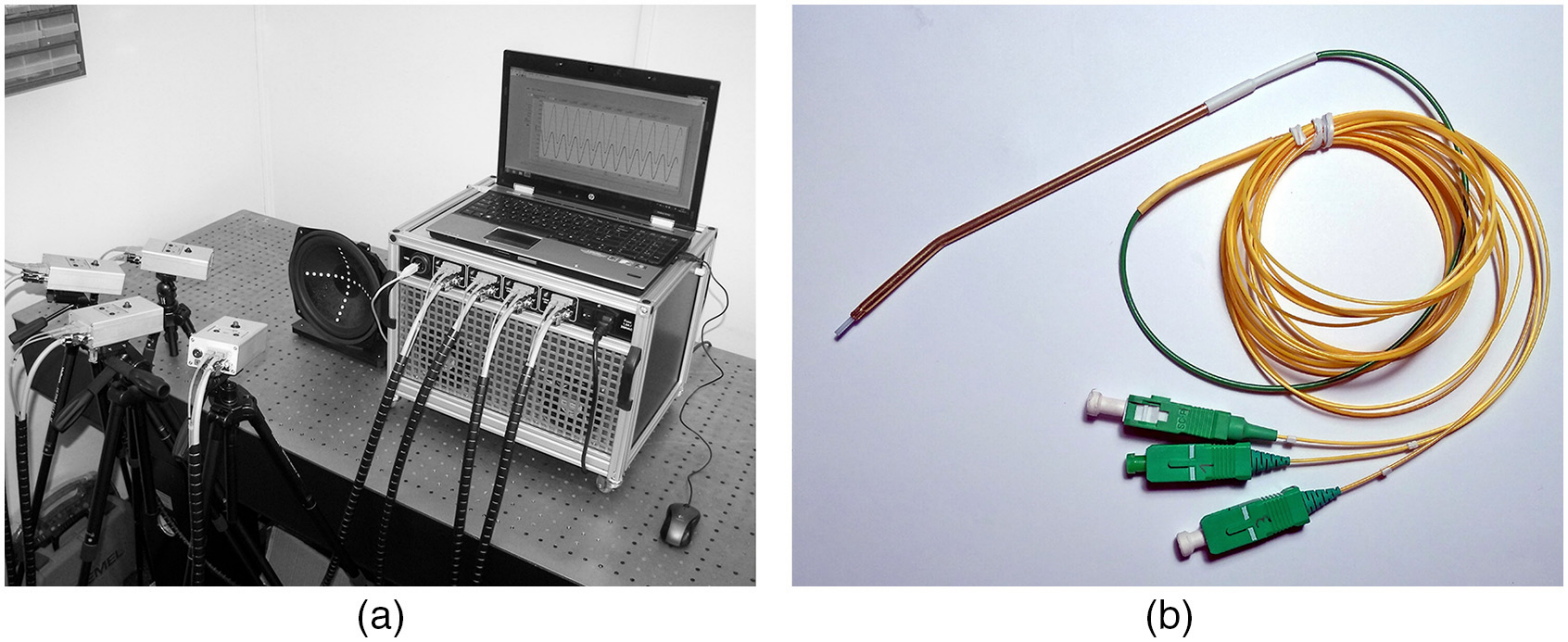
(a) The four channel laser-fiber Doppler vibrometer^47^ and (b) the handheld probe. The probe, consisting of a ceramic ferrule with the three optical fibers mounted inside a metal shielding sleeve, was connected to one of the four channels of the laser-fiber vibrometer. Two optical fibers are for the forward and return paths of the 1550 nm laser, and one is for the red navigation spot.

The displacement of the auditory ossicles was measured using a four-channel laser fiber Doppler vibrometer (LFDV) developed by Laser and Fiber Electronics Group at Wroclaw University of Science and Technology [Fig. 1(a)]. Detailed information on the operation of this device is presented in the articles by Wąż et al. (2009)^43^ and Wąż et al. (2014).^47^ The most important parameters of the LFDV are presented in Table 2.

**Table 2:** Parameters of the four-channels laser fiber Doppler vibrometer.^47^

Parameter	Value
Number of channels	4
Wavelengths of the analyzing radiation (nm)	1549.32, 1550.12, 1550.92, and 1551.72
Range of measured vibration speeds	0÷3 m/s
Frequency of measured vibrations	0.1 Hz÷500 kHz
Resolution of displacement measurements	10 pm
Dynamic range (displacement measurement) at 1 kHz	70 pm÷400 μm
Input optical power	−40÷10 dBm
Number of phase/frequency demodulators	1 × phase demodulator (displacement), 2 × FM demodulators (velocity)
Measuring distance	1 mm÷2.5 m (depending on the probe)
Noise	Fig. 2
Auxiliary laser radiation (observation of the analysis point)	635 nm (red)
Software for control and acquisition	LabView

The measuring probe consisted of a ceramic ferrule with an external diameter of 1.25 mm mounted inside a metal shielding sleeve [Fig. 1(b)]. Three standard single-mode telecommunications optical fibers with a jacket diameter of 125  μm were glued into the ferrule. The diameter of the measuring spot coming out of the optical fiber is a function of the distance between the probe tip and the object, and for 1.5 mm, it is about 300  μm. The diameter of the navigation spot was slightly larger because a telecommunications optical fiber at this wavelength (about 635 nm) is not single mode. The metal tube was bent at 7 deg with a 50-mm radius of the curvature.

**Fig. 2:**
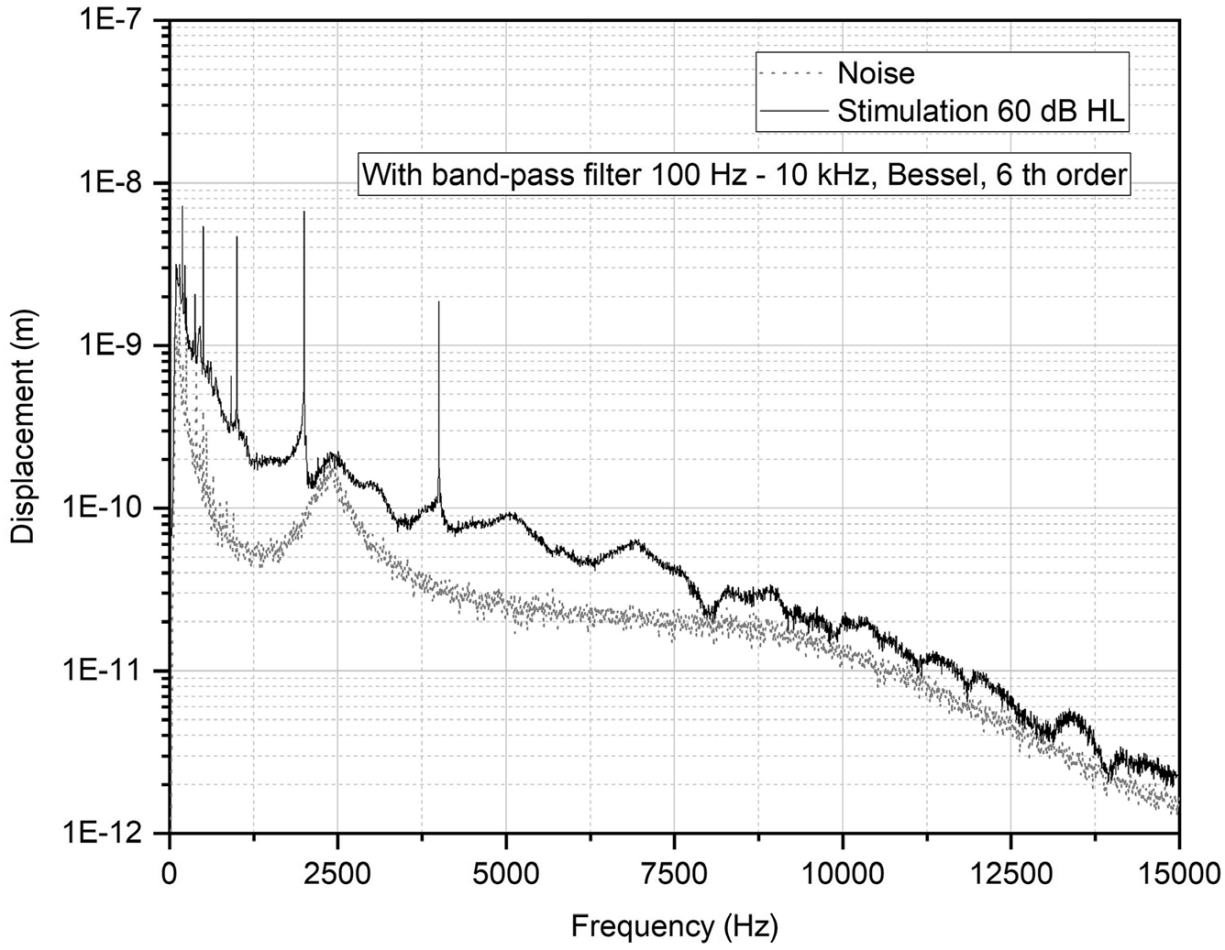
Noise floor (gray dots) measured on a stationary object and the displacement of the posterior crus of the stapes with a stimulation of 60 dB HL (black line). The probe was fixed on a tripod.

The response was recorded in sweeps of 200 ms. The quadrature demodulator as a displacement decoder was used.^47^ Sweeps exceeding the heuristically determined noise threshold were rejected from the analysis.^48^ The baseline-to-peak amplitude displacement was determined from a 6-s long-response signal consisting of 30 sweeps. Figure 2 shows an example of the data obtained during the measurements against a background noise.

A small piece of reflective tape was placed on the incus body and the posterior crus of the stapes. The measurement at each site was carried out three times. The probe was held by one of the two surgeons two times; the third time the probe was placed on a tripod. The intensities of all four tonal stimuli were equal and were set to 90 dB HL.

Measurements on the posterior crus of the stapes were also carried out in relation to the intensities of the sound stimuli. The intensities were decreased from 90 dB HL in 10 dB steps until no response was obtained. The probe was fixed on a tripod.

### 2.4 Statistical Analysis

The measurements were compared with data reported in the literature on the basis of confidence intervals on the difference between means. Multivariate analysis of variance (MANOVA) was carried out for stimulus frequency, bone, site, and fixation method (operators/tripod). Pearson correlation coefficients between the logarithm of the displacement and the stimulus intensity were determined.

## 3 Results

**Fig. 3:**
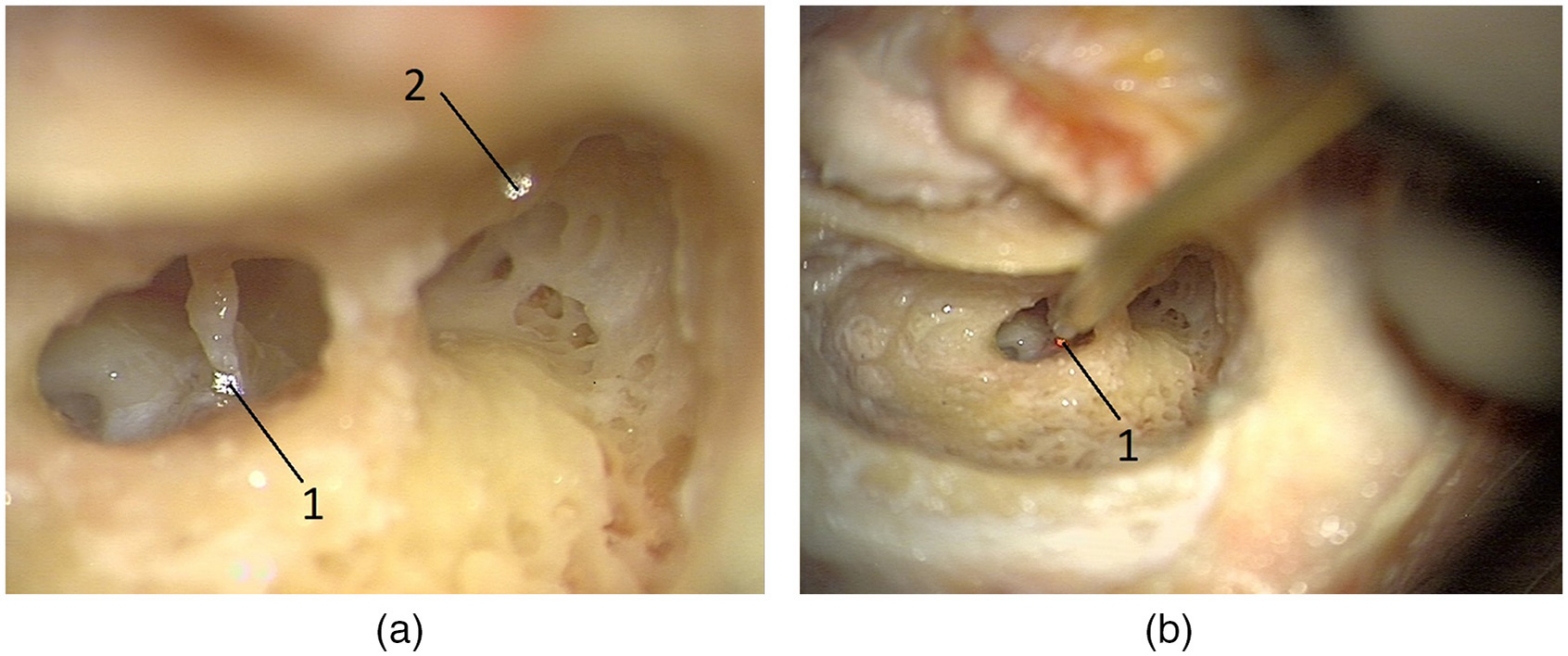
Photograph of the temporal bone with reflective material (a) placed at the measuring sites: on the posterior crus of the stapes (1) and the incus body (2). The approach to the tympanic cavity was performed by means of the standard antromastoidectomy followed by posterior tympanotomy with preservation of the facial nerve canal and the chorda tympani. (b) Photograph of the measurement on the posterior crus of the stapes (1). The probe was introduced into the tympanic cavity through a posterior tympanotomy.

In the period from 25.06.2019 to 23.01.2020, measurements were performed on 6 out of 7 temporal bones from 6 donors aged 48 to 68 years (median 62, SD 7). All of the ears appeared normal on otoscopic inspection; however, during mastoidectomy of the temporal bone no. 2, inflammatory changes were found in the tympanic cavity, and this bone was excluded from further analysis. A photograph of the temporal bone with reflective material at the sites and the second photo taken during the measurement on the posterior crus of the stapes are shown in Fig. 3.

**Fig. 4:**
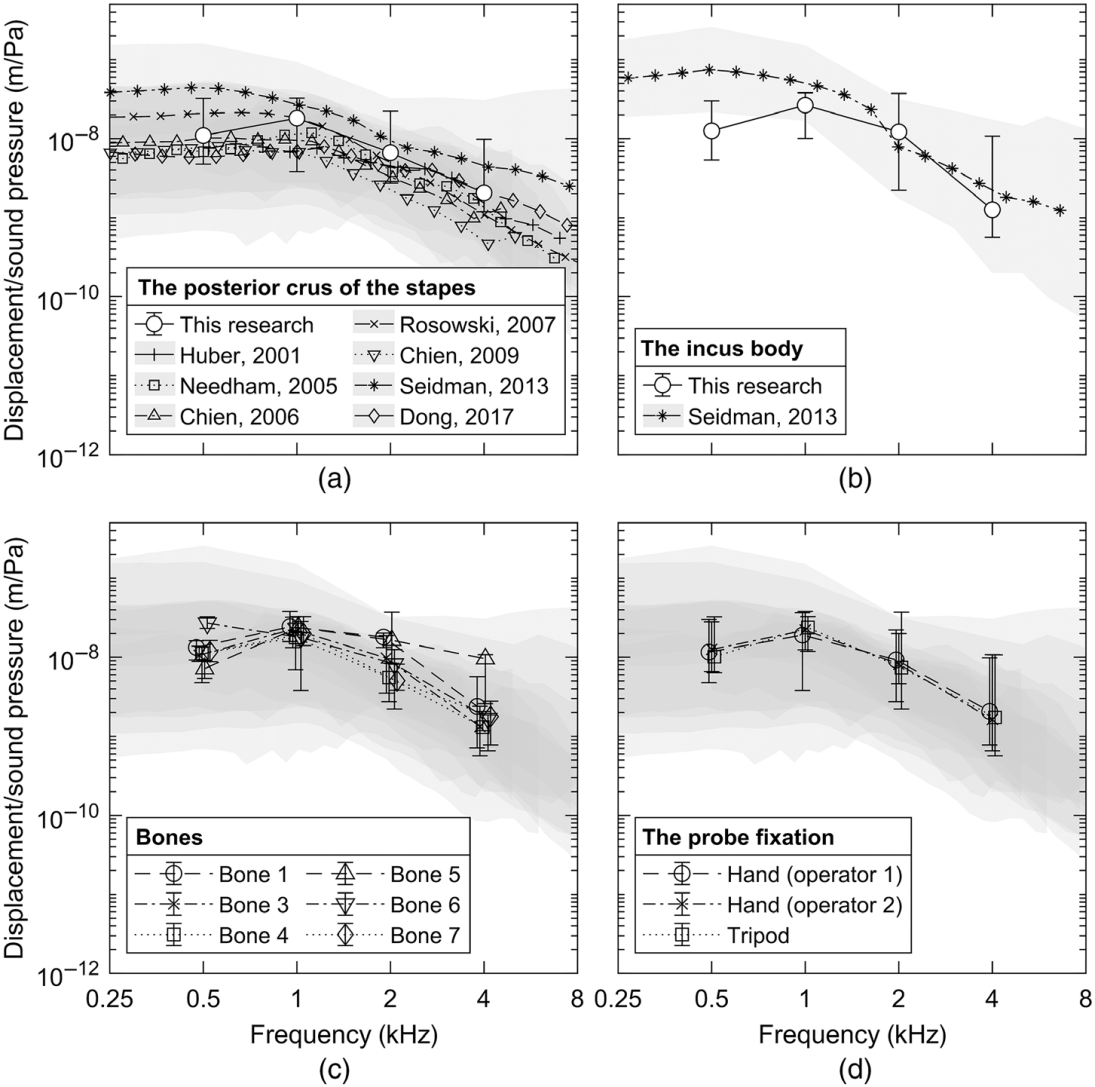
The middle ear transfer functions. Single data point represents the interpolated median of 18 measurements of the posterior crus of the (a) stapes and (b) incus body taken on six temporal bones by two surgeons and using a tripod. 95% confidence intervals determined using bootstrapping are marked with whiskers. The gray areas present 95% confidence intervals reported in the literature. A slight horizontal shift at the frequencies 500 Hz, 1, 2, and 4 kHz was introduced to improve symbol visualization. The article by Rosowski et al.^16^ presented an average of 13 other studies. The measurements in the research by Nedham et al.^2^ were carried out on the stapes head. The analogous chart for (c) temporal bone and (d) the fixation method is presented by means of the interpolated median of 6 and 12 (6 bones × 2 sites) measurements, respectively.

The middle ear transfer functions were calculated for measurements on the posterior crus of the stapes and the incus body as well as for individual temporal bones and regarding the probe fixation method (Fig. 4, Table S1 in the Supplementary Material). The results were compared with data reported in the literature.^1^^–^^3^^,^^12^^,^^16^^,^^17^^,^^31^ Statistically significant differences at p<0.05 were obtained only for the study by Seidman et al.^17^ and only for the measurement at 500 Hz on the incus body [Fig. 4(b)]. The difference between the means was at the level of 62.1  nm/Pa.

The data were subjected to MANOVA. Statistically significant differences were obtained for frequency ($F_{3,143}=29.37$, p<0.001, and $\eta^{2}=0.35$), bone ($F_{5,143}=4.61$, p=0.001, and $\eta^{2}=0.01$), and measurement site ($F_{1,143}=4.74$, p=0.03, and $\eta^{2}=0.02$) in the absence of statistically significant differences for the probe fixation method ($F_{2,143}=0.15$, p=0.862, and $\eta^{2}=0.001$). Standard deviations of the means were 6.9, 2.6, 1.9, and 0.6  nm/Pa for frequency, bone, site, and fixation, respectively. Test–retest reliability was assessed by means of intersurgeon differences. The standard deviation of the intersurgeon differences was determined at the level of 3.26  nm/Pa with a Cronbach alpha at 0.96 (95% CI 0.93 to 0.98).

**Fig. 5:**
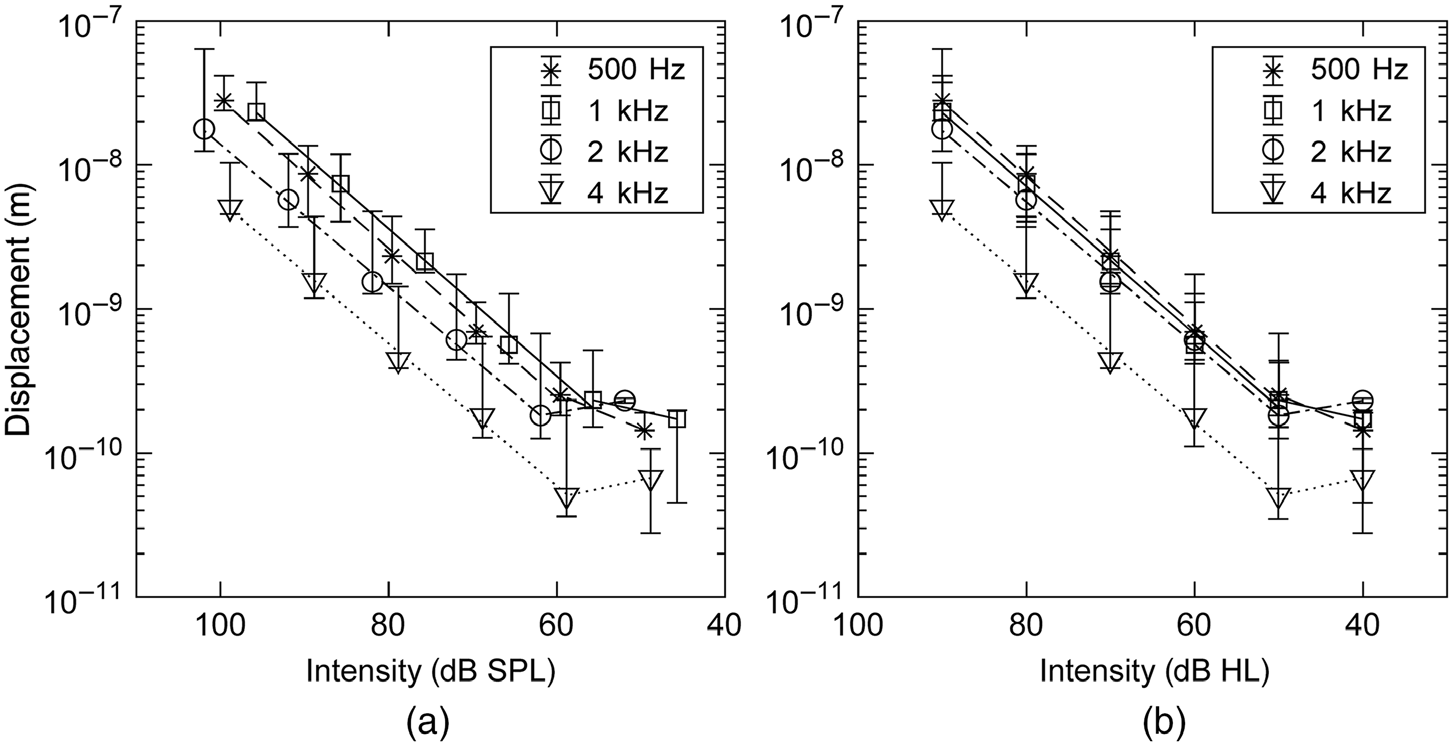
The displacement of the posterior crus of the stapes in relation to the intensity of the sound stimulus in (a) dB SPL and (b) dB HL. Single data point presents interpolated median of 6 measurements. Whiskers present 95% confidence intervals determined by means of bootstrapping.

The displacement of the posterior crus of the stapes in the relation to the stimulus intensity (Table S2 in the Supplementary Material) is shown in Fig. 5. The logarithm of the displacement decreases linearly with a decrease in the stimulus intensity in the range up to 50 dB HL. Pearson’s correlation coefficients were determined at the levels of 0.91, 0.94, 0.91, and 0.87 for the frequencies 0.5, 1, 2, and 4 kHz, respectively. The noise floor was present at the mean levels of 383 pm (SD 207), 251 pm (SD 43), 206 pm (SD 55), and 51 pm (SD 17) at frequencies 0.5, 1, 2, and 4 kHz, respectively.

## 4 Discussion

Measurements of the auditory ossicles displacement evoked by a sound stimulus were carried out by means of an HLFVP. The results were compared with data reported in the literature^1^^–^^3^^,^^12^^,^^16^^,^^17^^,^^31^ obtaining high consistency.

No statistically significant differences at the level of p<0.05 were obtained between hand and tripod measurement. This indicates that tremor has negligible influence on the results. The observation is in line with expectations as the physiological tremor ranging from 8 to 12 Hz^49^ is significantly different from the frequency of the ossicles vibration.

A linear relation between the displacement logarithm and the stimulus intensity was obtained at 50 dB HL and above. The threshold at 50 dB HL was found to be significantly higher than the value determined intraoperatively at 21.6 dB HL (SD 4.6).^21^ The difference may be related to the duration of the measurement as well as methodological differences in the assessment of the response threshold, which in the case of one study^21^ was evaluated subjectively in the time domain. However, the method of threshold assessment is of secondary importance due to the linearity of the process and the possibility of estimating the threshold from measurements at higher intensities.

**Fig. 6:**
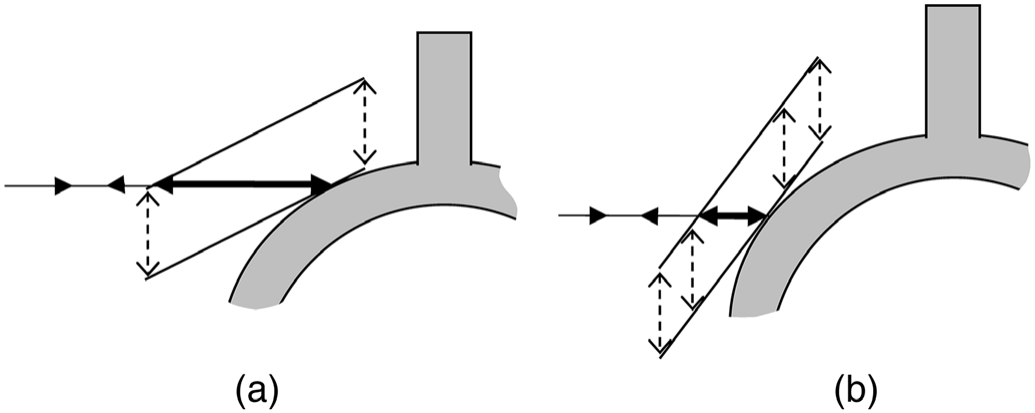
Effects of the angle between the laser beam and the reflecting surface on displacement measurements. Both figures show the laser beam (full arrow) forming a right angle with the direction of stapes displacement (dashed arrow). The measured displacement (bold arrow) is greater in (a) than (b) as it depends on the angle between the laser and the reflective surface.

The displacement measured by means of laser vibrometry is strongly dependent on the measuring angles between the laser beam and the vibration direction^31^ and the reflecting surface (Fig. 6). Controlling the angles is necessary to improve measurement uncertainty, especially since angle ranges for a handheld probe are wider than for a long-distance measurement. An alternative approach for measuring angles would be to standardize the site and the angles taking into account anatomical limitations and surgical convenience. Due to the difficulty of measuring angles in the operating room, standardization seems to be more efficient.

In this work, the measurement uncertainty may also be affected by the setup method of stimulus intensity. The sound pressure values at the tympanic membrane were determined on the REDD basis for the ER-3a with a foam ear-tip. This method is less precise than using an in-the-ear microphone such as ER-7, especially since the auricle was tilted forward and held in place by the self-retaining Weitlaner retractor, which could affect the ear canal geometry.

In this paper, the 1550-nm wavelength was used primarily because of compatibility with telecom, since optics and optical fibers for this wavelength are widespread and relatively inexpensive. At 1550 nm, light scattering on dry bone was sufficient for measurement. However, on cadaveric bones, incoming body fluids changed the scattering to reflectance, which is more directional, and made it difficult for the signal to reach the receiving system. Therefore, it was necessary to apply reflective tape. Optimizing the wavelength in terms of the reflectance of moist bone can contribute to improved measurement quality.

The tip of the measuring probe had a diameter of 1.25 mm. This dimension was related to the diameter of the shielding sleeve and the ferrule, into which the optical fibers were inserted. Theoretically, the diameter of the tip could be <300  μm and is limited by dimensions of the shielding sleeve for three optical fibers of 125  μm each. The minimum bending radius of the probe tip associated with optical fiber bending losses is estimated at about 10 mm.

## 5 Conclusions

The feasibility of using an HLFVP to measure the displacement of the auditory ossicles has been confirmed. HLFVP offers the possibility of carrying out measurements at various angles; however, this needs to be standardized taking into account anatomical limitations and surgical convenience. An additional advantage of the curved HLFVP tip in terms of access to the measurement site needs to be evaluated during the endoscopic approach.

## Code, Data, and Materials Availability

The raw data are presented in Tables S1 and S2 in the Supplementary Material.
