# Handheld laser-fiber vibrometry probe for assessing auditory ossicles displacement (Erratum)

**DOI:** 10.1117/1.JBO.26.7.079801

**Published:** 2021-07-30

**Authors:** Marcin Masalski, Adam Wąż, Przemysław Błauciak, Tomasz Zatoński, Krzysztof Morawski

**Affiliations:** a Wroclaw Medical University, Department of Otolaryngology Head and Neck Surgery, Wroclaw, Poland; b Wroclaw University of Science and Technology, Department of Biomedical Engineering, Wroclaw, Poland; c Wroclaw University of Science and Technology, Department of Field Theory, Electronic Circuits, and Optoelectronics, Wroclaw, Poland; d Wroclaw Medical University, Department of Neurosurgery, Wroclaw, Poland; e University of Opole, Institute of Medical Sciences, Opole, Poland

## Abstract

The erratum corrects an author affiliation.

This article [*J. Biomed. Opt.* **26**(7), 077001 (2021) doi: 10.1117/1.JBO.26.7.077001] was originally published on 21 July 2021 with erroneous affiliation listed for Tomasz Zatoński. The correct affiliation is Wroclaw Medical University, Department of Otolaryngology Head and Neck Surgery, Wroclaw, Poland.

The error was corrected, and the article was republished on 22 July 2021.

