# Supplementary material for: Handheld laser-fiber vibrometry probe for assessing auditory ossicles displacement
Source: J Biomed Opt. 2021 Jul 21;26(7):077001. doi: 10.1117/1.JBO.26.7.077001 (PMC8292735; doi:10.1117/1.JBO.26.7.077001)
Supplement: Supplementary file 1 [file JBO_026_077001_SD001.pdf]

Table S1 Baseline to peak displacement amplitudes measured with a handheld laser vibrometry probe (HLFVP) evoked by a 90 dB HL sound stimulus.

Measurement site	The posterior crus of the stapes				The incus body			
Frequency [Hz]	500	1000	2000	4000	500	1000	2000	4000
Measurement for Bone 1 [nm]								
Operator 1	49.8	79.7	81.0	7.0	39.1	34.2	78.2	17.6
Operator 2	54.9	82.5	88.5	7.8	41.4	37.4	78.8	14.8
Tripod	48.9	68.4	58.7	2.2	31.3	34.9	89.9	5.0
Measurement for Bone 3 [nm]								
Operator 1	38.4	56.7	73.3	3.3	39.8	41.3	32.2	7.4
Operator 2	30.0	43.9	54.2	2.9	46.9	55.0	23.4	4.9
Tripod	33.3	69.6	83.3	1.8	33.6	28.7	15.6	6.4
Measurement for Bone 4 [nm]								
Operator 1	43.9	54.4	37.3	4.3	16.2	15.1	12.1	3.7
Operator 2	43.8	50.1	20.4	3.8	32.1	27.0	26.7	4.3
Tripod	46.2	58.4	29.4	3.8	30.1	25.6	22.4	6.4
Measurement for Bone 5 [nm]								
Operator 1	15.2	34.5	44.6	6.7	28.1	70.9	98.5	30.4
Operator 2	25.7	56.3	58.7	27.9	22.5	48.3	36.9	23.3
Tripod	18.1	46.2	137.8	27.7	26.4	47.8	74.7	28.9
Measurement for Bone 6 [nm]								
Operator 1	85.1	18.2	37.3	4.8	88.0	8.3	36.9	5.3
Operator 2	65.9	51.1	46.2	1.7	95.1	47.7	35.7	5.1
Tripod	72.6	50.5	8.2	3.3	110.2	29.5	15.4	5.7
Measurement for Bone 7 [nm]								
Operator 1	38.3	40.5	26.2	2.4	51.2	41.5	18.2	8.6
Operator 2	29.8	30.5	24.3	5.0	55.4	44.6	20.4	6.4
Tripod	41.4	70.8	36.1	3.0	35.3	46.9	17.0	6.0

Table S2 Baseline to peak displacement amplitudes of the posterior crus of the stapes measured with a handheld laser vibrometry probe (HLFVP) at sound intensities from 90dB HL to 40 dB HL.

Stimulus intensity	Frequency [Hz]			
	500	1000	2000	4000
Measurement for Bone 1 [nm]				
90 dB HL	52.3	45.1	80.2	13.0
80 dB HL	17.1	14.9	15.0	5.5
70 dB HL	5.5	4.5	6.0	1.8
60 dB HL	1.4	1.6	2.2	0.40
50 dB HL	n/a	0.55	0.85	0.18
40 dB HL	n/a	0.25	0.28	0.04
Measurement for Bone 3 [nm]				
90 dB HL	33.6	28.7	15.6	6.4
80 dB HL	10.3	9.3	7.2	2.2
70 dB HL	2.6	2.7	1.6	0.56

60 dB HL	0.73	0.71	0.80	0.23
50 dB HL	n/a	0.19	0.34	0.08
40 dB HL	n/a	n/a	n/a	n/a
Measurement for Bone 4 [nm]				
90 dB HL	30.1	25.6	22.4	6.5
80 dB HL	5.4	5.1	3.0	1.8
70 dB HL	1.9	2.3	1.3	0.54
60 dB HL	0.54	0.52	0.56	0.18
50 dB HL	0.23	0.21	0.21	0.05
40 dB HL	0.18	0.06	n/a	n/a
Measurement for Bone 5 [nm]				
90 dB HL	44.0	107.3	84.3	29.5
80 dB HL	11.9	27.7	26.8	11.2
70 dB HL	3.7	9.3	10.9	3.5
60 dB HL	1.2	2.5	2.9	1.2
50 dB HL	0.35	0.75	0.98	0.41
40 dB HL	0.18	0.22	0.30	0.13
Measurement for Bone 6 [nm]				
90 dB HL	110.2	29.5	15.4	5.7
80 dB HL	32.5	8.8	4.7	2.0
70 dB HL	10.4	2.6	1.9	0.75
60 dB HL	3.2	0.71	0.64	0.23
50 dB HL	0.53	0.29	0.16	0.04
40 dB HL	0.24	n/a	n/a	n/a
Measurement for Bone 7 [nm]				
90 dB HL	35.3	46.9	17.0	6.0
80 dB HL	10.9	14.2	5.8	1.5
70 dB HL	2.9	3.8	1.6	0.49
60 dB HL	0.87	1.1	0.51	0.14
50 dB HL	0.29	0.29	0.15	0.05
40 dB HL	n/a	n/a	n/a	n/a
